# Radioimmunotherapy Targeting B7-H3 *in situ* glioma models enhanced antitumor efficacy by Reconstructing the tumor microenvironment

**DOI:** 10.7150/ijbs.87763

**Published:** 2023-08-15

**Authors:** Meng Zheng, Yan Wang, Fengqing Fu, Kaijie Zhang, Yanan Wang, Shandong Zhao, Qingfeng Liu, Huiwen Mu, Xueguang Zhang, Liyan Miao

**Affiliations:** 1 Department of Clinical Pharmacology, The First Affiliated Hospital of Soochow University, Suzhou, SZ, China.; 2 Institute for Interdisciplinary Drug Research and Translational Sciences, College of Pharmaceutical Sciences, Soochow University, Suzhou, SZ, China.; 3 State Key Laboratory of Radiation Medicine and Protection, Soochow University, Suzhou, SZ, China.; 4 Jiangsu Institute of Clinical Immunology, The First Affiliated Hospital of Soochow University, Suzhou, SZ, China.; 5 SuZhou Bright Scistar Antibody Biotech co., Ltd, 303-305, Bldg 15, NO.8, Jinfeng Road, Suzhou, SZ, China.

**Keywords:** B7-H3, radionuclide drug conjugate, glioblastoma, pharmacodynamics, tumor microenvironment.

## Abstract

Radionuclide drug conjugates (RDCs) with antibodies serve as a novel approach for the treatment of malignant tumors including glioblastoma. However, RDCs require optimal antibodies to work efficiently. Hu4G4, a novel B7-H3-targeting humanized monoclonal IgG1 antibody, is highly specific for the human B7-H3 protein (a marker of tumor cells, including glioblastoma cells). Herein, we established ^131^I-labeled hu4G4 (^131^I-hu4G4) and showed that it specifically bound to B7-H3 with high affinity (Kd = 0.99 ± 0.07 nM) and inhibited the growth of U87 cells *in vitro*. ^131^I-hu4G4 displayed potent *in situ* antitumor activity in a mouse model of glioma based on GL261 Red-Fluc-B7-H3 cells. More importantly, ^131^I-hu4G4 remodeled the tumor microenvironment and promoted the transformation of glioma from “cold” to “hot” tumors by promoting CD4^+^ and CD8^+^ T cell infiltration and the polarization of M2 to M1. Therefore, the antitumor activity observed with ^131^I-hu4G4, together with its ability to enhance antitumor immune responses, makes it a novel candidate for radioimmunotherapy of glioblastoma.

## Introduction

Glioblastoma multiforme (GBM) is the most aggressive brain cancer with a high chance of tumor recurrence (∼90%) 1, 2. Despite repeated attempts to enhance the treatment options, including PD-1/PD-L1-targeting checkpoint inhibition and CAR-T cell therapy, the clinical outcomes for individuals with glioblastoma have remained the same for many years. The likelihood of surviving for 2 years, even with optimum care, is less than 30% 3. Therefore, more effective therapeutic alternatives for reversing immune suppression against GBM tumors are urgently needed.

Some local therapies, such as radiotherapy, can eradicate most primary tumors and cause immunogenic cell death (ICD) to commence systemic anticancer immunotherapy as a “tumor *in situ* vaccine” 4-6. Radionuclide drug conjugates (RDCs) can deliver targeted radiation to disseminated cancer cells by means of systemically administering radiopharmaceuticals. Thus, unlike conventional radiotherapy, RDCs specifically affect cells expressing relevant molecular targets 7, which reduces dosage deposition in healthy tissues, even those close to a tumor mass. The effectiveness of RDC was demonstrated in non-Hodgkin's lymphoma when ^131^I was combined with an anti-CD20 antibody 8.

B7-H3, also known as CD276, is highly aberrantly expressed in a variety of tumor tissues, such as glioblastoma 9 and gastric cancer 10 tissues, but is rarely expressed in normal tissues. Recently, preclinical and clinical trials have been initiated for several monoclonal antibodies against B7-H3, including a bispecific antibody (B7-H3/PD-L1) 11, an antibody-drug conjugate (Ds-7300a) 12 and enoblituzumab (MGA271) 13. Furthermore, the limited toxicity of monoclonal antibodies targeting B7-H3, as demonstrated in clinical trials, suggests that CD276 is a rational target for RDC development 13, 14. Improvements in RDC technologies and preclinical successes suggest that opportunities exist for improving B7-H3-targeted therapy using RDCs, which may reinforce or complement B7-H3 targeting by immune-based agents.

Our previous research showed 15 that hu4G4, a novel B7-H3-targeting humanized monoclonal immunoglobulin (Ig)G1 antibody, has high targeting specificity for the human B7-H3 protein and exhibits excellent tumor targeting in mouse models of xenograft tumors based on U87 glioblastoma cells. In the present study, we prepared ^131^I-hu4G4 by radiolabelling it with the most widely used therapeutic radionuclide, ^131^I. The antibody-cell, interaction processes of ^131^I-hu4G4 were evaluated based on cellular uptake and retention *in vitro*. To determine the relative binding strength of the antibody to its target, competitive binding analysis was performed to elucidate the binding kinetics of the ligand and characterize its binding site. Subsequently, its pharmacological activity was assessed in U87 cells *in vitro* and in a mouse model of glioma involving GL261 Red-FLuc-B7-H3 cells *in vivo*. More importantly, the effect of ^131^I-hu4G4 on the tumor immune microenvironment (TIME) was also evaluated. The results of this study highlight the development of a novel RDC (^131^I-hu4G4), which represents a promising strategy for treating GBM and opens up interesting possibilities for improving T cell infiltration and the polarization of M2 to M1 macrophage in GBM.

## Materials and methods

### Reagents

Sodium chloride, sodium carbonate, citric acid, and sodium citrate were purchased from Sinopharm Chemical Reagent Co., Ltd. Glass fiber chromatographic paper was purchased from Agilent Technologies. Dulbecco's modified Eagle's medium (DMEM) and fetal bovine serum (FBS) were purchased from Gibco (USA). The Hematoxylin and Eosin Staining Kit was purchased from Solarbio (China). The CCK8 Kit was purchased from Dojindo (Kumamoto, Japan). Crystal violet and 4% paraformaldehyde were purchased from Beyotime Biotechnology (China). Donkey Anti-Rabbit IgG H&L (Alexa Fluor 488; catalog number ab150061), Donkey Anti-Rabbit IgG H&L (Alexa Fluor 594; catalog number ab150076), Donkey Anti-Mouse IgG H&L (Alexa Fluor® 488; catalog number ab150105), Donkey Anti-Mouse IgG H&L (Alexa Fluor® 594; catalog number ab150108), Donkey Anti-Rat IgG H&L (Alexa Fluor 594; catalog number ab150156), and Donkey Anti-Rat IgG H&L (Alexa Fluor® 488; catalog number ab150156) were purchased from Abcam (USA). Antibodies against HMGB1 (catalog number 10829-1-AP), CRT (catalog number 10292-1-AP), CD80 (catalog number 18704-1-AP), and CD206 (catalog number 10829-1-AP) were purchased from Proteintech (USA). Antibodies against CD4 (catalog number 14-0041-82), CD8α (catalog number 14-0081-82), and F4/80 (catalog number 14-4801-82) were purchased from eBioscience (USA). Blasticidin (catalog number R21001) was obtained from ThermoFisher (USA). All procedures involving mice and all experimental protocols were approved by the Institutional Animal Care Committee of Soochow University.

### Cell culture and tumor models

For cell culture, the human glioblastoma U87 cell line was provided by Suzhou Bright Scistar Biotechnology Co., Ltd. and grown in DMEM supplemented with 10% FBS and 1% penicillin‒streptomycin. The cells were kept in a humidified atmosphere with 5% CO_2_ at 37°C, and the medium was changed every other day. A 70-80% confluent monolayer was released using 0.1% trypsin and dissociated into a single cell suspension for subsequent cell culture. The Bioware® Brite Cell Line GL261 Red-FLuc cell was purchased form PerkinElmer, Inc. and overexpressed human B7-H3 through lentivirus using the standard protocol, which is named as GL261 Red-FLuc-B7-H3 cell. The culture method for the GL261 Red-FLuc-B7-H3 cell line was basically the same as that for the U87 cell line.

For the GL261 Red-FLuc-B7-H3 *in situ* glioma tumor mouse model, 40 male C57 BL/6 mice were implanted intracranially with 1 × 10^7^ cells suspended in 10 µL of PBS. Briefly, an intraperitoneal injection of 10% chloral hydrate (3 mL/kg) was used to anesthetize the mice. Mice were injected with 1×10^7^ GL261 Red-FLuc-B7-H3 cells suspended in 10 μL PBS through stereotactic injection. After inoculation, C57 BL/6 mice were placed in a specific pathogen free (SPF) animal house. On the 3^rd^ day after inoculation, tumors were photographed using the small animal live fluorescence imaging system to observe their size. Finally, C57 BL/6 mice were randomly divided into four groups (n=10): PBS, hu4G4, Na^131^I and ^131^I-hu4G4 groups. The experiment was conducted three times.

### Synthesis of ^131^I-hu4G4 and *In vitro* stability analysis

Hu4G4 was radiolabeled with ^131^I using the chloramine‑T method 16. The *in vitro* stability of ^131^I-hu4G4 in serum or saline was determined using the radio-iTLC method at various time intervals (24, 48, 72, 96, 144, and 168 h).

### *In vitro* experiments

CCK-8 assays were performed to assess cell viability. Briefly, U87 cells were seeded in 24-well plates (2 × 10^4^ cells/well) and exposed to various doses of hu4G4 (0, 6.4, 64, 320, 640, or 1280 ng/mL) for 48 h at 37°C. After adding CCK-8 solution (10 µL) to each well, the cells were incubated for an additional 1.5 h at 37°C. Absorbance values were measured at 450 nm using a microplate reader (Multiskan FC, Thermo Scientific). In addition, U87 cells viabilities at different ^131^I-hu4G4 concentrations were also investigated. To be specific, ^131^I-hu4G4 was added to the 24-well plates and incubated at 37 °C for 4 h at concentrations of 0.037, 0.37, 3.7 and 37 KBq/mL. Following incubation, the cells were washed three times with refrigerated PBS before being applied to DMEM supplemented with 10% FBS for 24, 48, or 72 h at 37°C. Cell viability was measured by CCK-8 solution at each time point.

To evaluate colony formation, 2000 U87 cells were plated in 6-well plates and treated for 4 h with hu4G4 (143 ng/mL), Na^131^I (55.5 KBq/mL), or ^131^I-hu4G4 (55.5 KBq/mL). After incubation at 37°C for 14 days, the cells were fixed in 4% paraformaldehyde and stained with crystal violet for 15 minutes at room temperature (25 °C). Subsequently, the colonies were then counted and the plates were photographed.

Wound-healing assays were performed to assess cell migration. U87 cells were planted in 6-well plates (1 × 10^6^ cells/well) and allowed to form confluent monolayers. Each wound was made with a pipette tip and treated with different groups (PBS, 55.5 KBq/mL Na^131^I, 143 ng/mL hu4G4, or 55.5 KBq/mL ^131^I-hu4G4) for 4 h. Images of the cells were taken after 0, 4, 12, and 24 h using a microscope (Olympus, Japan), and cell migration was assessed by measuring the gap sizes in several fields. The microscopic images were quantified using distance measurements.

Cell-cycle progression was also analyzed after treatment. 2 × 10^5^ U87 cells were seeded in 6-well plates and treated for 4 h with hu4G4 (143 ng/mL), Na^131^I (55.5 KBq/mL), PBS, or ^131^I-hu4G4 (55.5 KBq/mL). After incubation for 48 h at 37°C, the cells were collected, stained with PI/RNase, and analyzed using flow cytometry (Beckman CytoFLEX, USA) according to standard procedures.

For ICD assessments, 2 × 10^4^ U87 cells were seeded in 24-well plates and treated for 4 h with hu4G4 (143 ng/mL), Na^131^I (55.5 KBq/mL), PBS (10 μL), or ^131^I-hu4G4 (55.5 KBq/mL). The cells were then stained with antibodies (HMGB1 and CRT) and imaged using an Olympus microscope.

The methods for measuring the cellular uptake, binding affinity, and receptor saturation for ^131^I-hu4G4 are described in the Supplementary materials.

### *In vivo* experiments in an *in situ* mouse model of glioma tumors originating from GL261 Red-FLuc-B7-H3 cells

Each group of tumor mice was intracranially injected with 10 μL PBS, hu4G4 (0.15 mg/kg in 10 μL), Na^131^I (1.11 MBq/10 μL, 0.15 mg/kg), or ^131^I-hu4G4 (1.11 MBq/10 μL, 0.15 mg/kg) after GL261 Red-FLuc-B7-H3 cell implantation on days 0 and 3. At various time points, a PerkinElmer IVIS® spectroscope was used to track luciferase expression in tumor cells as a representative indicator of tumor growth. To exclude the interference from the method of injection, normal C57 BL/6 mice were injected with Na^131^I (1.11 MBq/10 μL, 0.15 mg/kg) or ^131^I-hu4G4 (1.11 MBq/10 μL, 0.15 mg/kg) through stereotactic injection at the same time.

To study the PK behavior of ^131^I-hu4G4 after intracranial injection, blood samples (~ 10-20 µL) were taken from each mouse's tail vein at intervals of 1, 12, 36, 48, or 72 h after intracranial administration These samples were weighed immediately and quantified with a γ-counter.

To assess the biodistribution of ^131^I-hu4G4 after intracranial injection *ex vivo* at the experimental endpoints, the major organs and tissues of each mouse were collected, including the brain, heart, liver, spleen, lungs, kidneys, stomach, pancreas, bones, joints, muscles, gonads, thyroid, and intestines. The tissues were immediately weighed and counted using a γ-counter, which enabled calculation of the %ID/g of each tissue sample.

Single-cell suspensions were derived from tumors for flow cytometric analysis. The cells were blocked with a CD16/CD32 antibody (1:200, BioLegend, 156604) after washing twice with PBS. Then, single-cell suspensions were stained with fluorescent dye-conjugated antibodies against CD3 (1:100, BioLegend, 100236), CD11b (1:200, BioLegend, 101206), CD8a (1:100, BioLegend, 100804), CD4 (1:100, BioLegend, 100568), F4/80 (1:200, BioLegend, 123110), CD206 (1:100, BioLegend, 141708), CD80 (1:100, BioLegend, 104714), or control IgG. The cells were analyzed using a CytoFLEX flow cytometer (Beckman Coulter, USA).

For H&E staining, tumors tissues were fixed with 4% paraformaldehyde. Then, tumors tissues dehydration was used with different alcohol concentration and embedded in paraffin and sectioned (thickness: 4 μm). The sections were then dewaxed in xylene and progressively rehydrated for 5 min using the following ethanol gradient: 100%, 100%, 95%, 90%, 80%, and 70%. According to the manufacturer's protocol, tumor sections were stained using H&E Staining Kits (Solarbio, China).

For immunofluorescence staining, the samples were processed as described in H&E staining. To access the CRT-expression profile, the tumor sections were incubated overnight at 4°C with an anti-CRT antibody (catalog number nb600-562, 1:200 dilution, Novus, USA), stained for 2 h at room temperature (25℃) with a secondary antibody, and then stained with DAPI. The images of specimens were recorded using a fluorescence microscope (BX53, Olympus). The fluorescence intensities of the resulting images were quantified and analyzed using Image Pro Plus software (version 6.0).

To assess the release of HMGB1, the tumor sections were permeabilized with Triton X-100 (0.5%, w/w) for 10 min before staining (using the same experimental process used for CRT staining). To evaluate T cell infiltration in brain tissues, T cell surface markers (CD4 and CD8α) were stained by IF staining. In the same manner, we investigated macrophage polarization in brain tissues via IF staining against macrophage surface markers (F4/80, CD80, and CD206), following the same experimental process used for CRT staining.

### Statistical analysis

For statistical analysis, SPSS (version 26.0) and GraphPad Prism (version 9) was used. The mean and standard deviation (SD) were determined to present the findings quantitatively, with all error bars indicating the SD. Student's *t*-test was performed and *p* < 0.05 were considered to significant differences.

## Results

### ^131^I-hu4G4 inhibits proliferation and migration and induces G2/M phase arrest and immunogenic cell death of U87 cells *in vitro*

We successfully labeled hu4G4 with ^131^I using different labeling processes** (Figure S1A). Figure S1B-1E** indicate that the specific activities of ^131^I-hu4G4 were 5.55 and 55.5 GBq/μmol in PBS and human serum, respectively, and were highly stable. ^131^I-hu4G4 showed high B7-H3 binding specificity and affinity in U87 cells in terms of cellular uptake, binding affinity, and receptor-saturation assays, which is important considering the binding characteristics of drug-target pharmacodynamic interactions required for RDCs to perform acceptably as drugs 17, 18 **(Figure S2)**.

**Figure 1:**
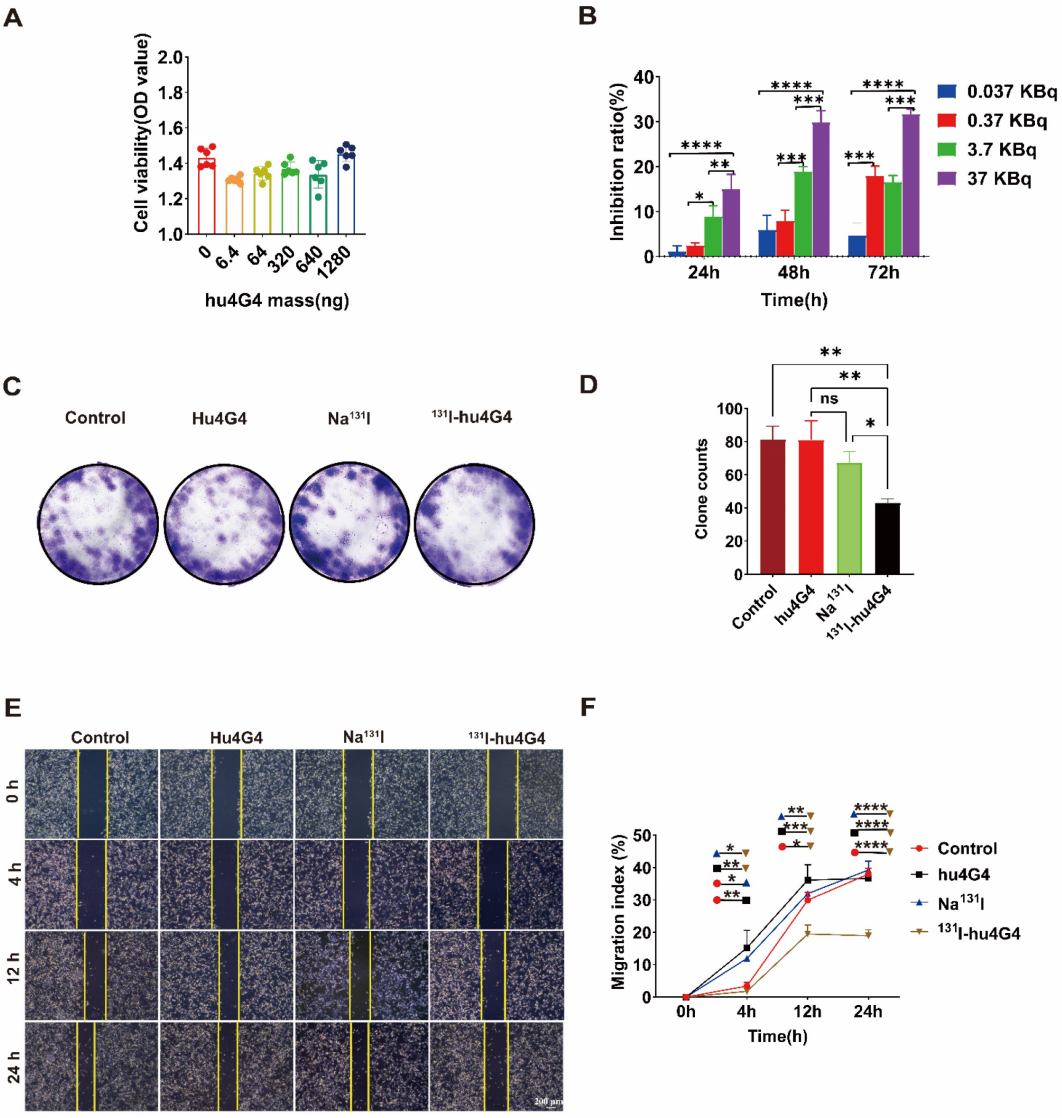
**^131^I-hu4G4 inhibited the viability of U87 cells *in vitro*.** (A) Effect of different hu4G4 antibody concentrations on U87 cell viability. (B) Effects of different radioactivity levels of ^131^I-hu4G4 on the viability of U87 cells at three time points (24, 48, and 72 h). (C) Images of U87 cells obtained in cell-cloning assays with each treatment group. (D) Quantitative results of cell-cloning assays found with U87 cells in each treatment group. (E) Images of U87 cells in cell-migration assays after each treatment. (F) Quantitative cell-migration data for U87 cells in each treatment group.

Based on the efficient uptake of ^131^I-hu4G4, we further assessed the cytotoxicity of hu4G4, ^131^I-hu4G4, Na^131^I, and PBS against tumor cells by conducting CCK-8 assays. In addition, colony-formation assays (plate format) were performed to examine U87 cell proliferation after treatment. After exposure to hu4G4 at doses ranging from 0 to 1,280 ng/mL, the viabilities of the U87 cells did not change significantly** (Figure 1A)**. However, ^131^I‑hu4G4 significantly inhibited U87 cell proliferation. This effect was time-dependent (24-72 h) and dose‑dependent** (0.037-37 KBq/mL; Figure 1B).** Furthermore, the colony-formation assays showed that ^131^I‑hu4G4 treatment resulted in fewer colonies than the blank control treatment **(Figure 1C, 1D)**. These findings showed that ^131^I-hu4G4 inhibited U87 cell proliferation.

The capability of tumor cells to move to distant places is essential for tumor metastasis. As shown in **Figure 1E,1F**, the wound-healing assay results proved that the percentages of wound closure in the PBS, Na^131^I, hu4G4, and ^131^I‑hu4G4 groups were 37.97 ± 1.85%, 36.65 ± 0.91%, 39.26 ± 2.73%, and 18.94 ± 1.82%, respectively, at 24 h after scraping.

The uncontrollable multiplication of cancer cells is caused by dysregulation of cell apoptosis or the cell cycle, which is reflected in the growth of the tumor. The transition from the G1/S to the G2/M phases of the cell cycle is the primary regulatory hurdle in this process. Flow cytometry was used to investigate the influence of ^131^I-hu4G4 on cell cycle distribution. ^131^I-hu4G4 dramatically raised the proportion of U87 cells in G2/M phase while decreasing the proportion of cells in G1 phase **(Figure S3)**, indicating that ^131^I-hu4G4 may promote cell G2/M phase arrest.

Irradiation induced ICD. Therefore, we investigated the capacity of ^131^I-hu4G4 to enhance CRT expression and HMGB1 release (two ICD biomarkers) in U87 cells **(Figure S4)**. U87 cells treated with PBS or hu4G4 alone showed modest intracellular CRT expression, whereas ^131^I-hu4G4 promoted CRT expression and accelerated the release of HMGB1 from the nuclei of U87 cells. These results demonstrate that ^131^I-hu4G4 functioned as an efficient ICD inducer.

### ^131^I-hu4G4 is mainly enriched in the brain in the GL261 Red-FLuc-B7-H3 *in situ* glioma tumor model after intracranial administration

The blood radioactive-uptake curves of mice at 1, 12, 36, 48, and 72 h after intracranial administration are shown in **Figure S5**. At 1 h after the first administration, the blood uptake of the ^131^I-hu4G4 group was 5.21 ± 2.82%ID/g, which was significantly lower than that of the Na^131^I group (24.82 ± 7.91%ID/g; *p* < 0.001). In addition, at 12 h after administration, the blood uptake of Na^131^I groups were 4.18 ± 2.75 %ID/g and that in ^131^I-hu4G4 group was 10.01 ± 9.02%ID/g, respectively. At 36 h after administration, the blood-uptake values of the ^131^I-hu4G4 and Na^131^I groups were less than 1%ID/g. The results showed that, compared with the Na^131^I group, the ^131^I-hu4G4 group showed less blood exposure after administration.

The biodistributions of ^131^I-hu4G4 and Na^131^I in a mouse orthotopic glioma model at the end of the experiment are shown in **Figure S6**. ^131^I-hu4G4 was mainly concentrated in the brain (total uptake in the brain including tumor tissues: 9.23 ± 5.24% ID/g), followed by the liver (1.57 ± 0.19%ID/g), spleen (1.41 ± 0.43%ID/g), kidneys (0.92 ± 0.28%ID/g), thyroid (0.54 ± 0.68%ID/g), and lungs (0.26 ± 0.22%ID/g). Other tissues showed less than 0.2% ID/g. Statistical analysis showed that the uptake of ^131^I-hu4G4 in the brain was significantly higher after intracranial administration than that in the liver (*p* < 0.01) and other tissues (*p* < 0.005; **Figure S6A**); thus, the exposure to radioactivity was significantly lower in other normal tissues. However, the uptake of Na^131^I was < 0.005%ID/g in all tissues, and no specific uptake was observed in any bodily tissues **(Figure S6B)**. These results were confirmed by situ glioma tumor-to-background ratios **(Figure S6E, 6F)**. The situ glioma tumor to heart, situ glioma tumor to liver, and situ glioma tumor to thyroid ratios in the ^131^I-hu4G4 group were significantly higher than those in the Na^131^I group (*p* < 0.05), and the brain: muscle ratio in ^131^I-hu4G4 group was significantly higher than that in the Na^131^I group (*p* < 0.01), as shown in **Figur**e** S6C,6D.**

### ^131^I-hu4G4 inhibits growth of orthotopic xenografts initiated from GL261 Red-FLuc-B7-H3 cells and induces immunogenic cell death in GL261 Red-FLuc-B7-H3 cells

**Figure 2:**
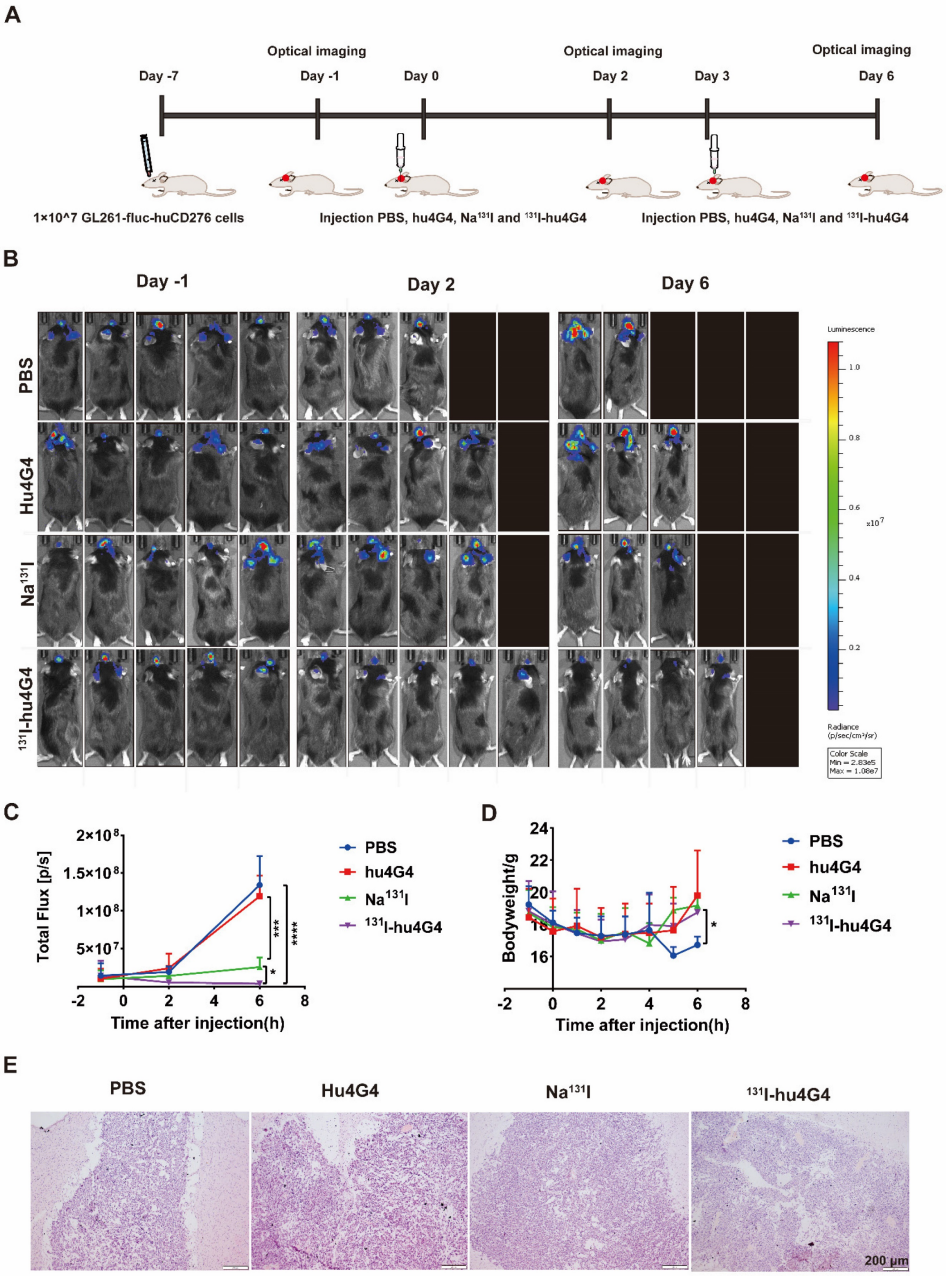
** The efficacy of ^131^I-hu4G4 in an *in situ* model of glioma (n = 5).** Luciferase expression (used as an indicator of tumor growth) was detected using a PerkinElmer IVIS® fluorescence-imaging device at 1 day before administration, 2 days after administration, and 6 days after administration. (A) Schematic representation of the treatment methods. (B) Intracranial fluorescence imaging of mice in the PBS, hu4G4, Na^131^I, and ^131^I-hu4G4 groups at 1 day before administration, 2 days after administration, and 6 days after administration. (C) Intracranial tumor growth in mice in the PBS, hu4G4, Na^131^I, and ^131^I-hu4G4 groups was analyzed before and after treatment by bioluminescence imaging. (D) Body-weight changes of mice in the PBS, hu4G4, Na^131^I, and ^131^I-hu4G4 groups were monitored during treatment. (E) H&E staining of tumor tissues in the PBS, hu4G4, Na^131^I, and ^131^I-hu4G4 groups at 6 days after the first administration (scale bars: 200 μm).

The antitumorigenic activity of ^131^I-hu4G4 was tested *in vivo* using GL261 cells transfected with a plasmid encoding Red-FLuc-B7-H3 to generate a mouse xenograft model of GBM **(Figure 2A)**. Using the small animal live fluorescence imaging system, we had selected 20 mice in the best condition for PD study (five mice/group). As shown in Figure **2B, 2C**, the therapeutic efficacy of ^131^I-hu4G4(total flux: 3.80×10^6^ ± 2.65×10^6^) was better than that of the hu4G4 group (total flux 1.19 × 10^8^ ± 2.74 × 10^8^) (*p* < 0.001) and the Na^131^I group (total flux: 2.57 × 10^7^ ± 1.26 × 10^7^) (*p* < 0.05) at day 6 after the first administration, while there was no difference between the hu4G4 and PBS groups. The therapeutic effect of each group corresponds to the mouse survival rate. At the same time, we observed that mice treated with PBS or hu4G4 alone demonstrated rapid tumor growth, with some mice dying beginning on the 2nd day after treatment **(Figure 2B,2C)**. The survival rate in the ^131^I-hu4G4 group was 80%, while that in the PBS, hu4G4, and Na^131^I groups was 40%, 60%, and 60%, respectively. In addition, no obvious changes in body weight were observed during the observation period, indicating that our strategy had no significant toxic effects on the mice **(Figure 2D)**. H&E staining showed that the brain tumor tissues were intact without necrosis **(Figure 2E)**.

**Figure 3:**
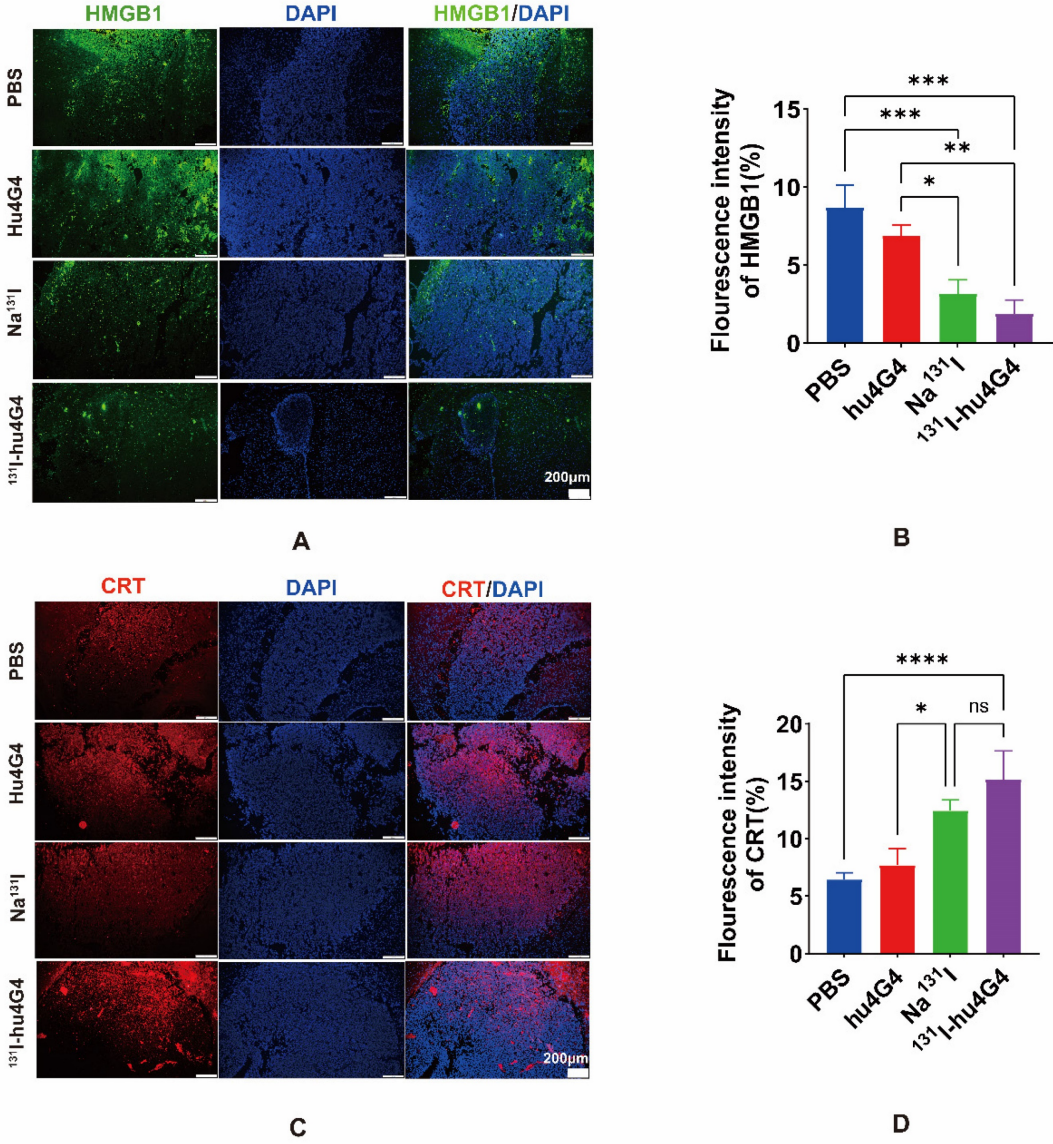
**^131^I-hu4G4 induces immunogenic cell death in an *in situ*** glioma tumor** (n = 3).** (A, C) immunofluorescence-staining images showing the HMGB1and CRT levels in brain glioma sections of mice in the different treatment groups. (B, D) Semi-quantitative analysis HMGB1 and CRT.

Some chemotherapeutics and/or ionizing radiation trigger ICD, which stimulates anti-tumor immune responses in a variety of malignancies. Thus, in order to evaluate the ICD profile caused by ^131^I-hu4G4 therapy, cell-surface CRT expression and nuclear HMGB1 expression were examined. In contrast to PBS treatment, ^131^I-hu4G4 treatment significantly upregulated cell-surface CRT expression while reducing nuclear HMGB1 expression, which was indicative of increased nuclear release of HMGB1 **(Figure 3)**. These results indicate that ^131^I-hu4G4 can potentially enhance tumor immunogenicity.

### ^131^I-hu4G4 promotes transformation of glioma from "cold" to "hot" tumors by reconstructing tumor microenvironment

**Figure 4:**
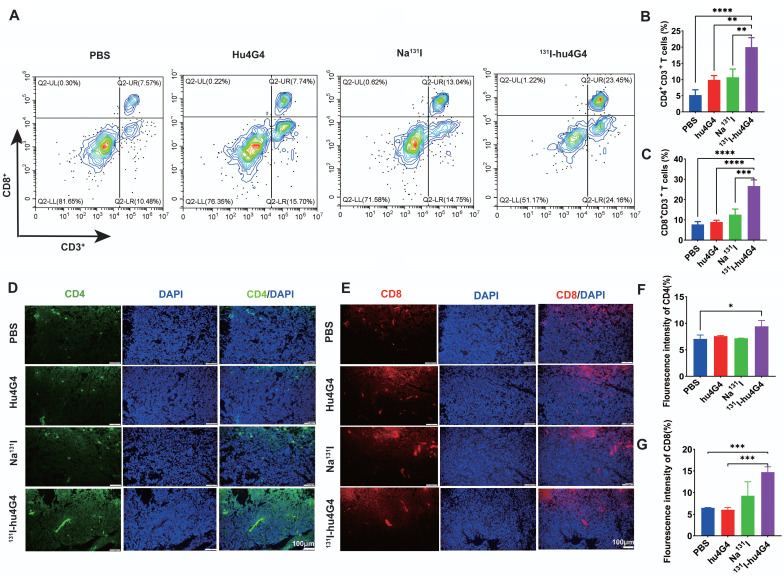
**^131^I-hu4G4 enhances T-cell infiltration into GBM tumors.** Tumors were surgically removed seven days following treatment. Tumor-derived single-cell suspensions were stained with CD3, CD8, CD4 antibodies, then flow cytometry was performed. Tumor sections were stained by CD8, CD4 antibodies. (A) Representative flow cytometry plots (gated on CD3^+^ CD8^+^ T cells. (B) and (C) Quantification of proportions of CD4^+^, CD8T^+^ CD3^+^ T cells. (D, E) Immunofluorescence-staining images showing the CD4 and CD8 levels in brain glioma sections from mice in different treatment groups. (F, G) Fluorescence intensities of CD4 (F)and CD8 %(G).

The mechanism underlying the excellent antitumor therapeutic efficiency of ^131^I-hu4G4 was evaluated by collecting primary tumors from mice on day 6 post-treatment and testing for tumor infiltrating lymphocytes using flow cytometry **(Figure 4A-4C)**. The tumors treated with ^131^I-hu4G4 possessed more activated CD8^+^ cytotoxic T lymphocytes among the tumor-infiltrating lymphocytes (26.69 ± 3.01 %) than those treated with PBS (7.75 ± 1.42 %), hu4G4 (8.90 ± 0.84 %), or Na^131^I (12.58 ± 2.74 %), as shown in **Figure 4A, 4B**. The ratios of CD4^+^ T and CD8^+^ T cells were similar, showing the most significant increases after ^131^I-hu4G4 treatment (20.00 ± 2.92 %) compared with those after PBS (5.21 ± 1.66 %) or hu4G4 (9.90 ± 1.37 %) treatment **(Figure 4C)**. Immunofluorescence analysis of tumor sections showed similar results. In addition, the average number of IFN γ^+^/CD3^+^ cells were significantly lower than that in the control group **(Figure S9)**.

**Figure 5:**
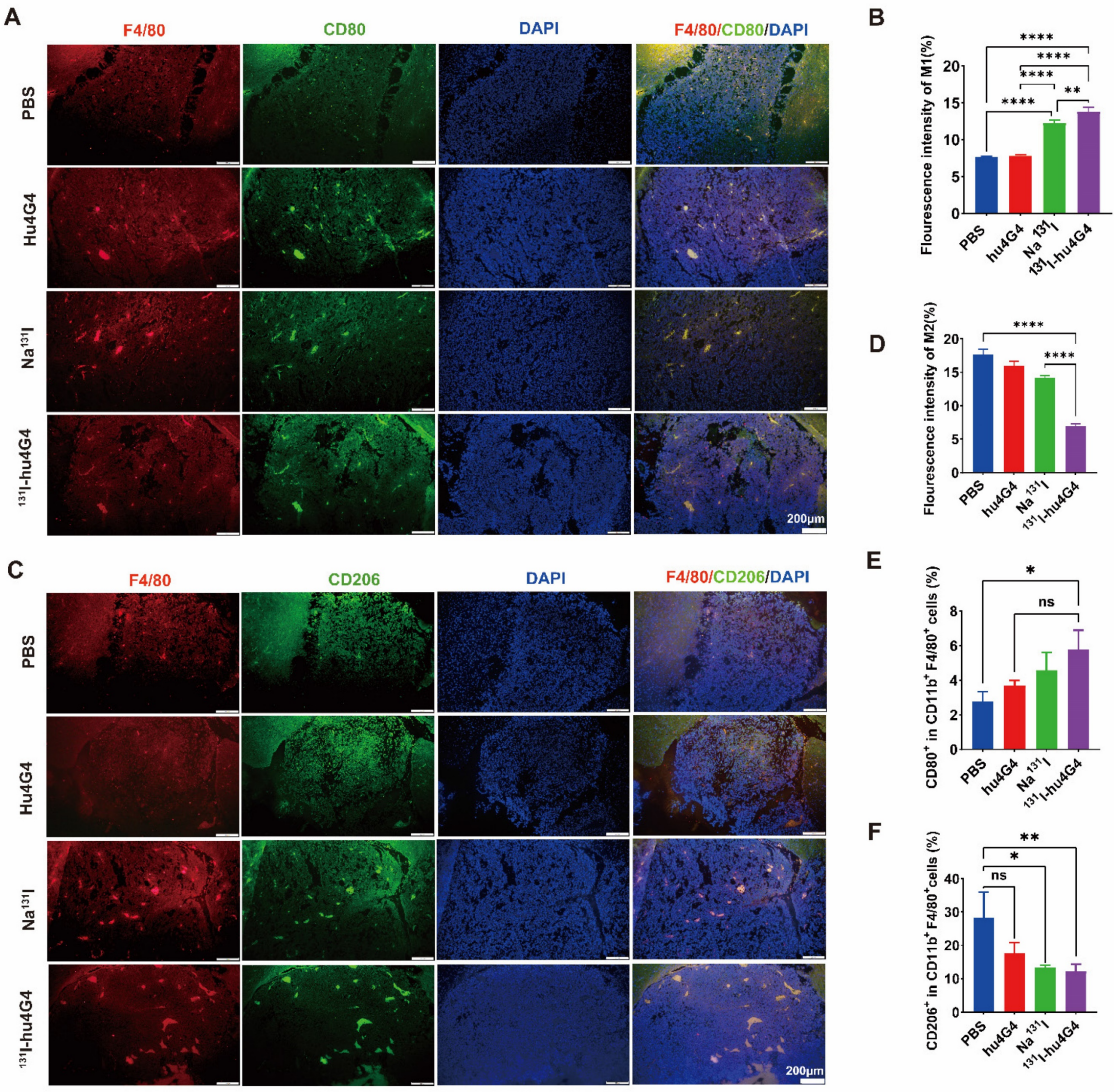
**^131^I-hu4G4 directs tumor-associated macrophages (TAMs) toward a pro-inflammatory phenotype that orchestrates tumor immunological responses.** Tumor-derived single-cell suspensions were stained with CD11b, F4/80, CD80, CD206 antibodies, then flow cytometry was performed. Tumor sections were stained by F4/80, CD80, CD206 antibodies. (A, C) Immunofluorescence-staining images showing the CD80^+^/F4/80^+^ M1 and CD206^+^/F4/80^+^ M2 levels in brain glioma sections from mice in different treatment groups. (B, D) Fluorescence intensities (%) of CD80^+^/F4/80^+^ M1(B) and CD206^+^/F4/80^+^ M2(D). Changes in the abundances of M1 macrophages (E), M2 macrophages (F) in the glioma microenvironment were also detected by flow cytometry. (E) Quantitative analysis of CD80^+^/F4/80^+^ M1 cells and (F) CD206^+^/F4/80^+^ M2 cells by flow cytometry.

Microglia reside in the central nervous system and exhibit a plastic phenotype that depends on microenvironmental conditions. Thus, we investigated whether ^131^I-hu4G4 can regulate microglial polarization in the GBM microenvironment. We found that the abundance of M1 microglia was increased by ^131^I-hu4G4 treatment, as the ratio of F4/80^+^ CD80^+^ cells was higher after ^131^I-hu4G4 treatment (5.80 ± 1.01 %) than after PBS (2.80 ± 0.55 %) or hu4G4 (3.68 ± 0.32%) treatment **(Figure 5E)**. In contrast, ^131^I-hu4G4 tended to reduce the abundance of M2 microglia, as the ratio of F4/80^+^ CD206^+^ cells in tumor tissues was significantly lower after ^131^I-hu4G4 treatment (12.18 ± 2.11 %) than after PBS treatment (28.27 ± 7.689 %) **(Figure 5F)**. Similar results were observed in immunohistochemical analysis **(Figure 5A-5D)**.

## Discussion

GBM is typically resistant to T cell-based immunotherapies which is consistent with its immunologically cold nature caused by an extraordinarily immunosuppressive microenvironment. Therefore, to treat GBM, we developed a novel RDC that specifically targets B7-H3.

The necessity for a therapeutic RDC is evident in light of radiation-induced necrosis in normal brain tissue and the related irreparable damage. ^131^I is widely used in clinical practice for cancer treatment and emits a decay energy of 971 keV, which is rapidly followed by β-decay and then γ-decay 19-21. Electrons penetrate only 0.6-2 mm of tissue, causing a low degree of damage to the healthy tissue surrounding a tumor. This suggests that hu4G4 labeled with ^131^I has great potential as an effective, low-toxicity treatment for gliomas. Radiolabeling processes must be quick, effective, repeatable, and affordable when preparing RIT agents. It is commonly known that direct radioiodination occurs 21. In this study, hu4G4 was labeled with ^131^I using the chloramine-T method 16 and different labeling processes. The results demonstrated that ^131^I-hu4G4 was successfully produced, had high specific activity (up to 55.5 GBq/mol), and was exceptionally stable in both human serum and PBS (RCP > 90%). Taking into account the quantity of antibody and the radioactive dose necessary for follow-up experiments, 5.55 GBq/μmol of specific activity was utilized for the ^131^I-labeled antibody.

For RDCs, the two main components of protein-cell interaction processes are cellular uptake 19 and cellular retention 22-24. *In vitro* cellular-uptake experiments showed that the rapid cellular uptake of ^131^I-hu4G4 was effectively blocked by exogenous hu4G4, suggesting that hu4G4 has binding-site specificity. **Figure S2B** shows a positive linear relationship between cell uptake and the number of cells involved, with a higher number of B7-H3-specific receptors leading to a higher cellular-uptake ratio. These results further indicated that labeling did not influence the ability of hu4G4 to bind specifically to B7-H3. A high binding affinity between the monoclonal antibody and the target antigen is another prerequisite for targeting tumor antigens* in vivo*. ^131^I-hu4G4 showed a high binding affinity for B7-H3 expressed in U87 cells, with an IC_50_ value of 1.83 ± 0.48 nM. The suitability of the tumor cell-surface biomarker as a target for medical imaging and therapy was evaluated using the amount of receptors expressed on the cell surface 25. The binding characteristics of the interactions between ^131^I-hu4G4 and the B7-H3 antigen on the surface of U87 cells provided a basis for conducting subsequent efficacy experiments.

*In vitro* cytotoxicity data revealed that ^131^I-hu4G4 specifically killed U87 cells. The CCK8 and plate colony-formation experiments revealed that ^131^I-hu4G4 had a significant effect on U87 cell proliferation. According to Shan et al 26, ^131^I-hu4G4 treatment caused cell cycle arrest in the G2/M phase, primarily due to ^131^I-decay, which triggered apoptosis and impeded U87 cell migration. This treatment strategy can extend the reach of radiation into tumor tissues, prevent radioactive harm to surrounding tissues, and reduce the risk of relapse. However, the efficacy of ^131^I-hu4G4 in glioma therapy yet to be determined *in vivo*.

Glioma treatment is fraught with challenges. In addition to the difficulty in completely removing lesions, most drugs and contrast agents cannot gain access to tumors through the blood-brain barrier 27, 28. Therefore, direct intracranial administration has been developed as a method to bypass the blood-brain barrier. During treatment, *ex vivo* pharmacokinetics and biodistribution experiments were conducted. As shown in **Figure S5**, after intracranial injection of Na^131^I and ^131^I-hu4G4, we found that 1 h after the first administration, the blood-intake value of the Na^131^I group (24.82 ± 7.90%ID/g) was significantly higher (*p* < 0.001) than that of ^131^I-hu4G4 group (5.21 ± 2.82%ID/g). Thus, we inferred that after intracranial injection, ^131^I-hu4G4 was mainly concentrated in the situ glioma tumor tissues, whereas the radioactive uptake in the blood was mainly caused by the drug entering the peripheral circulatory system along with the cerebrospinal fluid.

In addition, *ex vivo* biodistribution results showed that ^131^I-hu4G4 was highly enriched in the situ glioma tumor tissues, whereas the uptake in peripheral organs such as the liver and spleen was low. The observed enrichment of ^131^I-hu4G4 in the brain was mainly because B7-H3 was highly expressed in the tumor tissues. According to ^131^I-hu4G4 biodistribution, the uptake of ^131^I-hu4G4 in liver and spleen was significantly higher than that in other normal tissues. This is most likely brought on by a combination of elevated blood flow and IgG receptor IIIa binding to the Fc fragment. In addition, excessive tracer concentration in normal liver tissue may be caused by catabolism 29, as IgG is expelled after being removed via the liver. The presence of abnormal radioimmunoconjugates in non-target organs like the spleen is caused, according to Sharma et al.'s research 30, by the interaction of Fc gamma receptors expressed in the monocytes/macrophages that are common in the spleen. To exclude interference from the method of injection, normal C57 BL/6 mice were injected with Na^131^I or ^131^I-hu4G4 through stereotactic injection. Almost no radioactivity was observed in the brain **(Figure S7).** Furthermore, Na^131^I showed almost no peripheral uptake due to its rapid metabolic elimination after entering the peripheral circulatory system through the cerebrospinal fluid. Therefore, intracranial administration of ^131^I-hu4G4 can significantly increase the target: background ratio (up to 184 ± 105% in terms of the situ glioma tumor: muscle ratio), thus greatly reducing the radioactive uptake in normal tissue. Therefore, we consider that the method of injection has no effect on radioactivity uptake *in situ* glioma tumors. These results also confirm that the intracranial administration of ^131^I-hu4G4 is a good delivery method and provides a basis for the high efficacy of ^131^I-hu4G4 and relatively minor damage to normal tissues.

ICD is a type of regulated cell death driven by cellular stressors, which would strike the release of damage-associated molecular pattern (DAMP). Cells suffering ICD have the potential to function as both preventive and therapeutic anticancer vaccinations. As typical damage-associated molecular patterns, CRT and HMGB1^31^-^37^ have both been shown to be able to recruit DCs to the tumor site and subsequently encourage their maturation into prime adaptive antitumor immunity. Radiotherapy promotes antitumor immunity by inducing an adaptive immunological response driven by CD8^+^ T cells 6, 38, 39. When ICD is active, HMGB1 is actively released through two models. One way includes stimulating target cells, which causes HMGB1 to be secreted into the extracellular space 40. The second method entails the packing of HMGB1 into vesicles inside the cell (like lysosome), followed by the release of HMGB1 outside the cell 41, 42. In this study, increased cell-surface CRT expression and release of HMGB1 from the cell nucleus into the extracellular space indicated that ^131^I-hu4G4 therapy against gliomas elicited glioma cell ICD, which enhanced the tumor immunogenicity. The activation of both cytotoxic CD8^+^ T cells and CD4^+^ T helper cells is required for induction of an efficient antitumor immune response. Consistently, in this study, more activated CD8^+^ and CD4^+^ T were observed after ^131^I-hu4G4 therapy through both flow cytometry and immunohistochemistry. These results suggest that ^131^I-hu4G4 first elicited glioma cell ICD and reshaped the tumor microenvironment, which promoted the transformation of glioma from a “cold” to “hot” tumors. Tumor-associated macrophages comprise a heterogeneous population that includes resident microglia, brain-resident immune cells, and bone marrow-derived macrophages 43-45, which can become polarized to acquire an M2-like phenotype by factors within the immunosuppressive TIME. Ionizing radiation, according to the literature reports 46, 47, affects macrophage plasticity and drives tumor-associated macrophages toward a pro-inflammatory phenotype that mastermind tumor immunological responses. Our results showed that the polarization of tumor-associated macrophages into M2-like cells was reduced by ^131^I-hu4G4 therapy, indicating that ^131^I-hu4G4 therapy inhibited tumor cell growth and aggressiveness by regulating the polarization of microglia in the GBM microenvironment.

It should be mentioned that some limitations have existed in our study. GL261 is one of the most commonly used mouse brain tumor models for immunological and gene therapeutic investigations 48, 49. However, it is somewhat immunogenic. Increased MHCI or B7 costimulatory molecules expressed on their surface make them more sensitive to T cell recognition. This study did not consider the effect of the immunogenicity of GL261 cells on the change in TIME in brain tumors. Therefore, more mice tumor models are needed to verify the change in TIME.

In conclusion, we developed a novel radiolabeled, humanized antibody specific to the B7-H3 antigen, ^131^I-hu4G4. The antibody showed excellent binding specificity and affinity for the B7-H3 antigen and exhibited potent antitumor activity in an *in situ* model of glioma and enhanced antitumor immune responses. ^131^I-hu4G4 is a promising candidate for radioimmunotherapy against glioblastoma.

## Supplementary Material

Supplementary figures.

## References

[1] Alexander BM, Cloughesy TF (2017). Adult Glioblastoma. J Clin Oncol.

[2] Bi WL, Beroukhim R (2014). Beating the odds: extreme long-term survival with glioblastoma. Neuro Oncol.

[3] Norden AD, Wen PY (2006). Glioma therapy in adults. Neurologist.

[4] Kroemer G, Galluzzi L, Kepp O, Zitvogel L (2013). Immunogenic cell death in cancer therapy. Annu Rev Immunol.

[5] Krysko DV, Garg AD, Kaczmarek A, Krysko O, Agostinis P, Vandenabeele P (2012). Immunogenic cell death and DAMPs in cancer therapy. Nat Rev Cancer.

[6] Galluzzi L, Vitale I, Warren S, Adjemian S, Agostinis P, Martinez AB (2020). Consensus guidelines for the definition, detection and interpretation of immunogenic cell death. J Immunother Cancer.

[7] Gill MR, Falzone N, Du Y, Vallis KA (2017). Targeted radionuclide therapy in combined-modality regimens. Lancet Oncol.

[8] Chow VA, Rajendran JG, Fisher DR, Appelbaum FR, Cassaday RD, Martin PS (2020). A phase II trial evaluating the efficacy of high-dose Radioiodinated Tositumomab (Anti-CD20) antibody, etoposide and cyclophosphamide followed by autologous transplantation, for high-risk relapsed or refractory non-hodgkin lymphoma. Am J Hematol.

[9] Tang X, Zhao S, Zhang Y, Wang Y, Zhang Z, Yang M (2019). B7-H3 as a Novel CAR-T Therapeutic Target for Glioblastoma. Mol Ther Oncolytics.

[10] Kim NI, Park MH, Lee JS (2020). Associations of B7-H3 and B7-H4 Expression in Ductal Carcinoma *In situ* of the Breast With Clinicopathologic Features and T-Cell Infiltration. Appl Immunohistochem Mol Morphol.

[11] Aggarwal C, Prawira A, Antonia S, Rahma O, Tolcher A, Cohen RB (2022). Dual checkpoint targeting of B7-H3 and PD-1 with enoblituzumab and pembrolizumab in advanced solid tumors: interim results from a multicenter phase I/II trial. J Immunother Cancer.

[12] Yamato M, Hasegawa J, Maejima T, Hattori C, Kumagai K, Watanabe A (2022). DS-7300a, a DNA Topoisomerase I Inhibitor, DXd-Based Antibody-Drug Conjugate Targeting B7-H3, Exerts Potent Antitumor Activities in Preclinical Models. Mol Cancer Ther.

[13] Loo D, Alderson RF, Chen FZ, Huang L, Zhang W, Gorlatov S (2012). Development of an Fc-enhanced anti-B7-H3 monoclonal antibody with potent antitumor activity. Clin Cancer Res.

[14] Powderly J, Cote G, Flaherty K, Szmulewitz RZ, Ribas A, Weber J (2015). Interim results of an ongoing Phase I, dose escalation study of MGA271 (Fc-optimized humanized anti-B7-H3 monoclonal antibody) in patients with refractory B7-H3-expressing neoplasms or neoplasms whose vasculature expresses B7-H3. Journal for ImmunoTherapy of Cancer.

[15] Fu F, Zheng M, Zhao S, Wang Y, Huang M, Chen H (2023). Therapeutic Characterization of 131I-Labeled Humanized Anti-B7-H3 Antibodies for Radioimmunotherapy for Glioblastoma. Engineering.

[16] Hussien H, Goud AA, Amin AM, El-Sheikh R, Seddik U (2011). Comparative study between chloramine-T and iodogen to prepare radioiodinated etodolac for inflammation imaging. Journal of Radioanalytical and Nuclear Chemistry.

[17] Huber W (2005). A new strategy for improved secondary screening and lead optimization using high-resolution SPR characterization of compound-target interactions. J Mol Recognit.

[18] Onell A, Andersson K (2005). Kinetic determinations of molecular interactions using Biacore-minimum data requirements for efficient experimental design. J Mol Recognit.

[19] Kayano D, Kinuya S (2018). Current Consensus on I-131 MIBG Therapy. Nucl Med Mol Imaging.

[20] Sgouros G, Kolbert KS, Sheikh A, Pentlow KS, Mun EF, Barth A (2004). Patient-specific dosimetry for 131I thyroid cancer therapy using 124I PET and 3-dimensional-internal dosimetry (3D-ID) software. J Nucl Med.

[21] Zhang X, Liu DS, Luan ZS, Zhang F, Liu XH, Zhou W (2018). Efficacy of radioiodine therapy for treating 20 patients with pulmonary metastases from differentiated thyroid cancer and a meta-analysis of the current literature. Clin Transl Oncol.

[22] Adams DJ, Wahl ML, Flowers JL, Sen B, Colvin M, Dewhirst MW (2006). Camptothecin analogs with enhanced activity against human breast cancer cells. II: Impact of the tumor pH gradient. Cancer Chemother Pharmacol.

[23] Braun AH, Stark K, Dirsch O, Hilger RA, Seeber S, Vanhoefer U (2005). The epidermal growth factor receptor tyrosine kinase inhibitor gefitinib sensitizes colon cancer cells to irinotecan. Anticancer Drugs.

[24] Miao Y, Hoffman TJ, Quinn TP (2005). Tumor-targeting properties of 90Y- and 177Lu-labeled alpha-melanocyte stimulating hormone peptide analogues in a murine melanoma model. Nucl Med Biol.

[25] Hulme EC, Trevethick MA (2010). Ligand binding assays at equilibrium: validation and interpretation. Br J Pharmacol.

[26] Wu S, Ma K, Qiao WL, Zhao LZ, Liu CC, Guo LL (2018). Anti-metastatic effect of 131I-labeled Buthus martensii Karsch chlorotoxin in gliomas. Int J Mol Med.

[27] Wang C, Wu B, Wu Y, Song X, Zhang S, Liu Z (2020). Camouflaging Nanoparticles with Brain Metastatic Tumor Cell Membranes: A New Strategy to Traverse Blood-Brain Barrier for Imaging and Therapy of Brain Tumors. Advanced Functional Materials.

[28] Yin Y, Fu C, Li M, Li X, Wang M, He L (2016). A pH-sensitive hyaluronic acid prodrug modified with lactoferrin for glioma dual-targeted treatment. Mater Sci Eng C Mater Biol Appl.

[29] Shah DK, Betts AM (2013). Antibody biodistribution coefficients: inferring tissue concentrations of monoclonal antibodies based on the plasma concentrations in several preclinical species and human. MAbs.

[30] Sharma SK, Chow A, Monette S, Vivier D, Pourat J, Edwards KJ (2018). Fc-Mediated Anomalous Biodistribution of Therapeutic Antibodies in Immunodeficient Mouse Models. Cancer Res.

[31] Casares N, Pequignot MO, Tesniere A, Ghiringhelli F, Roux S, Chaput N (2005). Caspase-dependent immunogenicity of doxorubicin-induced tumor cell death. J Exp Med.

[32] Galluzzi L, Buque A, Kepp O, Zitvogel L, Kroemer G (2017). Immunogenic cell death in cancer and infectious disease. Nat Rev Immunol.

[33] Apetoh L, Ghiringhelli F, Tesniere A, Obeid M, Ortiz C, Criollo A (2007). Toll-like receptor 4-dependent contribution of the immune system to anticancer chemotherapy and radiotherapy. Nat Med.

[34] Garg AD, Krysko DV, Vandenabeele P, Agostinis P (2016). Extracellular ATP and P(2)X(7) receptor exert context-specific immunogenic effects after immunogenic cancer cell death. Cell Death Dis.

[35] Garg AD, Krysko DV, Vandenabeele P, Agostinis P (2012). Hypericin-based photodynamic therapy induces surface exposure of damage-associated molecular patterns like HSP70 and calreticulin. Cancer Immunol Immunother.

[36] Shen F, Feng L, Zhu Y, Tao D, Xu J, Peng R (2020). Oxaliplatin-/NLG919 prodrugs-constructed liposomes for effective chemo-immunotherapy of colorectal cancer. Biomaterials.

[37] Zhu Y, Yang Z, Dong Z, Gong Y, Hao Y, Tian L (2020). CaCO(3)-Assisted Preparation of pH-Responsive Immune-Modulating Nanoparticles for Augmented Chemo-Immunotherapy. Nanomicro Lett.

[38] Rapoport BL, Anderson R (2019). Realizing the Clinical Potential of Immunogenic Cell Death in Cancer Chemotherapy and Radiotherapy. Int J Mol Sci.

[39] Li X (2018). The inducers of immunogenic cell death for tumor immunotherapy. Tumori.

[40] Bonaldi T, Talamo F, Scaffidi P, Ferrera D, Porto A, Bachi A (2003). Monocytic cells hyperacetylate chromatin protein HMGB1 to redirect it towards secretion. EMBO J.

[41] Lu B, Wang C, Wang M, Li W, Chen F, Tracey KJ (2014). Molecular mechanism and therapeutic modulation of high mobility group box 1 release and action: an updated review. Expert Rev Clin Immunol.

[42] Pisetsky DS (2014). The expression of HMGB1 on microparticles released during cell activation and cell death *in vitro* and *in vivo*. Mol Med.

[43] Goldmann T, Wieghofer P, Jordao MJ, Prutek F, Hagemeyer N, Frenzel K (2016). Origin, fate and dynamics of macrophages at central nervous system interfaces. Nat Immunol.

[44] Li Q, Barres BA (2018). Microglia and macrophages in brain homeostasis and disease. Nat Rev Immunol.

[45] Sorensen MD, Dahlrot RH, Boldt HB, Hansen S, Kristensen BW (2018). Tumour-associated microglia/macrophages predict poor prognosis in high-grade gliomas and correlate with an aggressive tumour subtype. Neuropathol Appl Neurobiol.

[46] Wu Q, Allouch A, Paoletti A, Leteur C, Mirjolet C, Martins I (2017). NOX2-dependent ATM kinase activation dictates pro-inflammatory macrophage phenotype and improves effectiveness to radiation therapy. Cell Death Differ.

[47] Klug F, Prakash H, Huber PE, Seibel T, Bender N, Halama N (2013). Low-dose irradiation programs macrophage differentiation to an iNOS(+)/M1 phenotype that orchestrates effective T cell immunotherapy. Cancer Cell.

[48] Szatmari T, Lumniczky K, Desaknai S, Trajcevski S, Hidvegi EJ, Hamada H (2006). Detailed characterization of the mouse glioma 261 tumor model for experimental glioblastoma therapy. Cancer Sci.

[49] Genoud V, Marinari E, Nikolaev SI, Castle JC, Bukur V, Dietrich PY (2018). Responsiveness to anti-PD-1 and anti-CTLA-4 immune checkpoint blockade in SB28 and GL261 mouse glioma models. Oncoimmunology.

