# Supplementary material for: Radioimmunotherapy Targeting B7-H3 in situ glioma models enhanced antitumor efficacy by Reconstructing the tumor microenvironment
Source: Int J Biol Sci. 2023 Aug 15;19(13):4278–90. doi: 10.7150/ijbs.87763 (PMC10496502; doi:10.7150/ijbs.87763)
Supplement: Supplementary file 1 — Supplementary figures. [file ijbsv19p4278s1.pdf]

Supplementary Materials

Radioimmunotherapy Targeting B7-H3 in situ glioma models enhanced antitumor efficacy by Reconstructing the tumor microenvironment

1 Supplementary materials

1.1. Cell uptake, binding affinity and receptor saturation assays

For cell uptake, 2.5×10^5 U87 cells were seeded in 12-well plates to form a confluent monolayer. On the following day, U87 cells were washed three times with 0.01 M PBS (pH 7.4) and incubated with 1 mL serum-free DMEM for 2 h at 37°C. Then, 3.7×10^{-2} MBq ^{131}I-hu4G4 was added to each well and treated for 10 min 30 min, 1 h, 2 h, 4 h and 6 h at 37°C. The nonspecific binding of ^{131}I-hu4G4 to U87 cells was determined through coincubation with 100X unlabeled hu4G4. At each time point, 1 M 0.5 mL NaOH was used to separate cells for 5 min after the cells were washed three times with cold PBS. A gamma counter was used to measure the radioactivity in the cells, which was then corrected for physical decay. GraphPad Prism was used to analyze the data, and all trials were done in duplicate.

For binding affinity, 5×10^5 U87 cells were seeded in 6-well plates one day before the experiment. Cells were washed three times with 0.01 M PBS (pH 7.4) and incubated with 3.7×10^{-2} MBq ^{131}I-hu4G4 in 1 mL serum-free DMEM for 2 h at 37°C. Nonspecific binding of ^{131}I-hu4G4 with U87 cells was determined through coincubation with unlabeled hu4G4 (0.001-1000 nM final concentration). The remaining experimental procedures were the same as for the cellular uptake experiments.

For the receptor saturation assay, 1×10^5 U87 cells were seeded in 6-well plates one day before the experiment. Cells were washed three times with 0.01 M PBS (pH 7.4) and incubated with ^{131}I-hu4G4 (0.0625-1 nM final concentration) in 1 mL serum-free DMEM for 4 h at 37°C. Nonspecific binding of ^{131}I-hu4G4 with U87 cells was determined through coincubation with 100 times excess (0.6 μM) of hu4G4. After incubation, the cells were then separated with 1 M 0.5 ml NaOH for 5 min after three rounds of washing with chilled PBS. A gamma counter was used to measure the radioactivity in the cells. Specific binding (SB) = total binding (TB) - nonspecific binding (NSB). The data were analyzed with GraphPad Prism was used to analyze the data, and all trials were done in duplicate. A 1-site-fit

binding curve was used to obtain the dissociation constant (KD value) of ^{131}I-hu4G4. All experiments were carried out in triplicate.

2 Supplementary Figures

Figure S1.

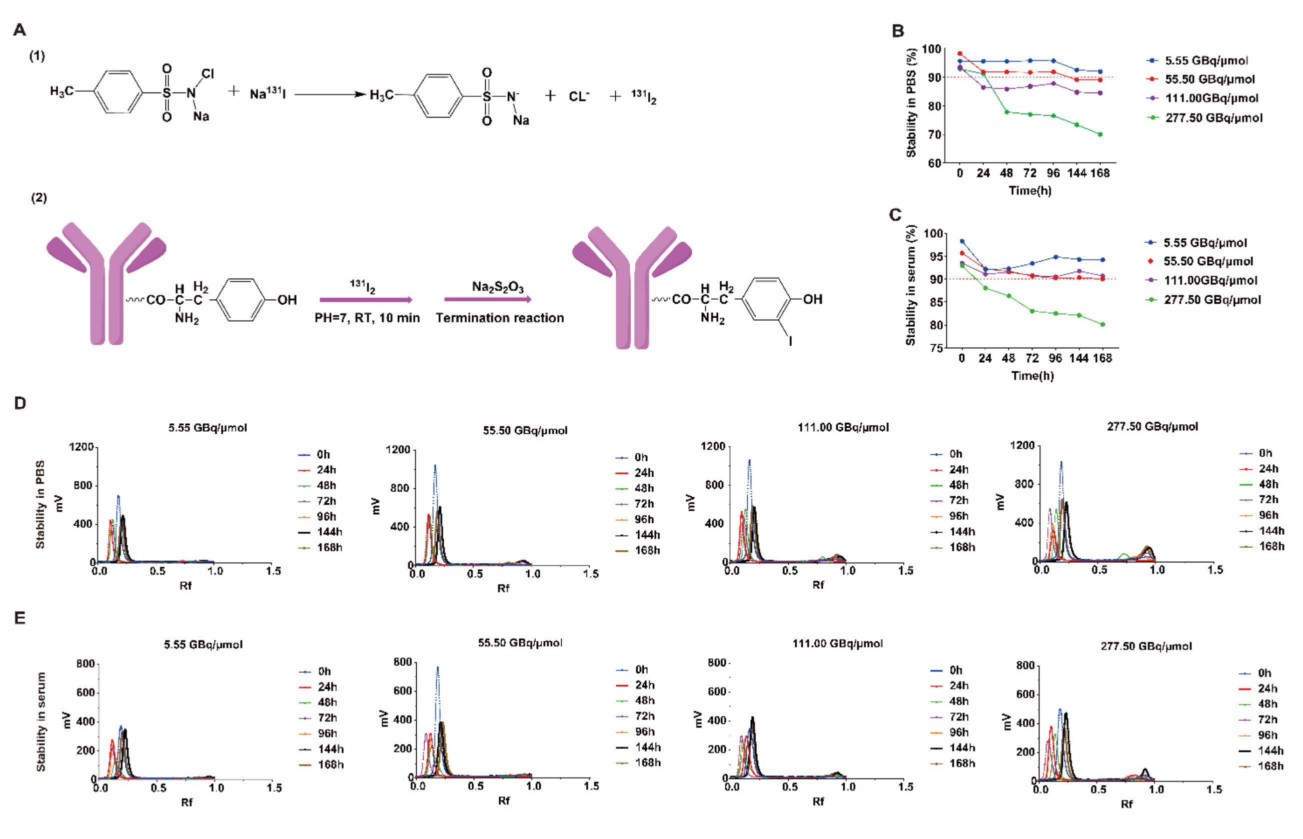

Figure S1. Hu4G4 was successfully labeled with ^{131}I, and 55.5 GBq/µmol was an optimal specific activity for labelling in vitro. (A) Scheme used to label hu4G4 with ^{131}I. (B, C) The quantitative analysis about stability of ^{131}I-hu4G4 labeled using four specific activities (5.5, 55.5, 111, and 277.5 GBq/µmoL) in PBS (B) and human serum (C) was assessed by radio-TLC analysis at different times (0, 24, 48, 72, 96, 144, and 168 h). (D, E) The map shown represent the radio-iTLC results of ^{131}I-hu4G4 in PBS (D) and human serum (E) at different times (0, 24, 48, 72, 96, 144, and 168 h).

Figure S2.

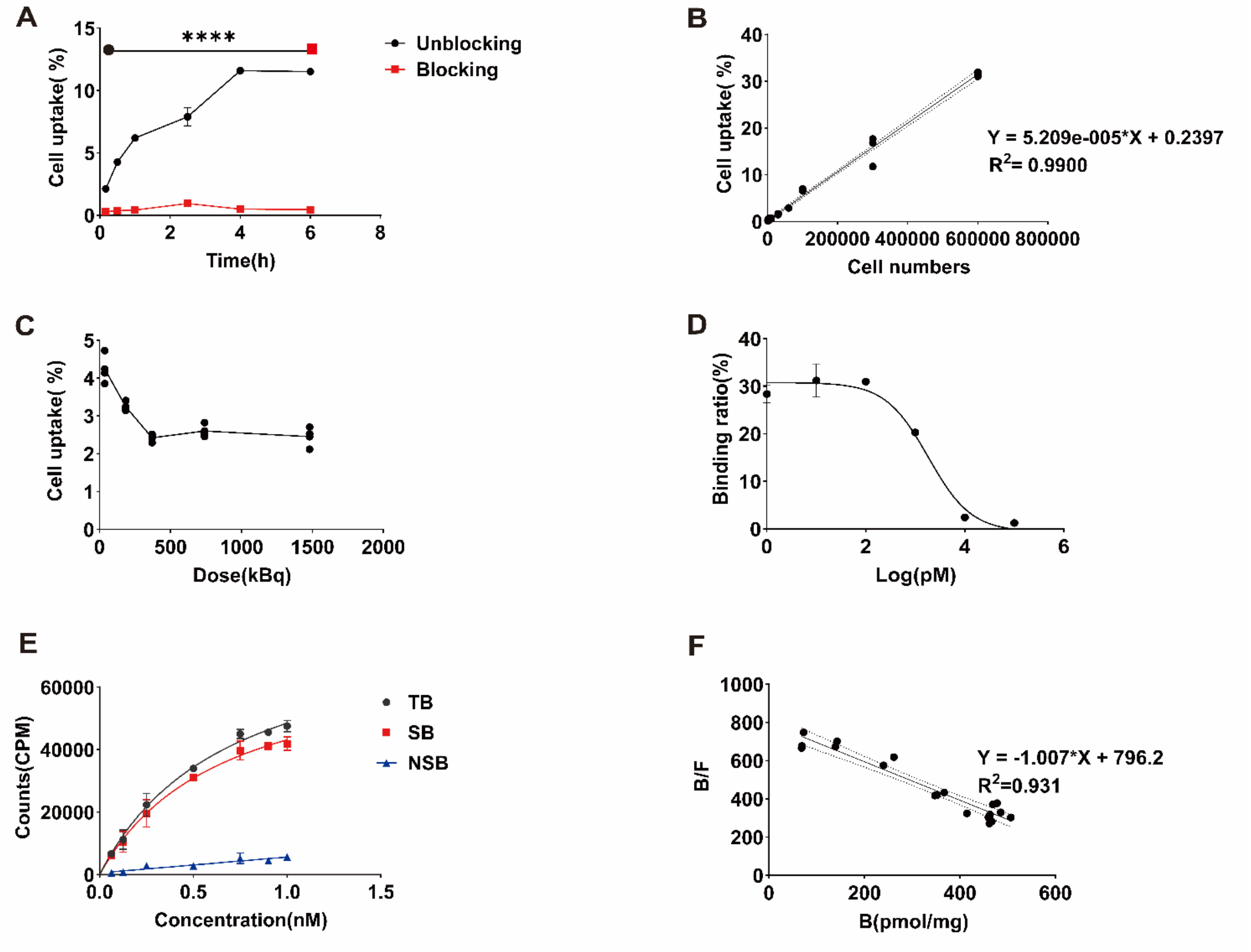

Figure S2. The ^{131}I-hu4G4 antibody showed high B7-H3-binding specificity and affinity in experiments performed with U87 cells. (A) ^{131}I-hu4G4 uptake by U87 cells at different time points. (B) 131I-hu4G4 uptake by different numbers of U87 cells. (C) U87 cell uptake of 131I-hu4G4 after labeling with different radioactivities. (D) Affinity of hu4G4 for U87 cells. (E) Total binding (TB), specific binding (SB), and non-specific binding (NSB) values of U87 cells treated with different concentrations of ^{131}I-hu4G4. (F) Scatchard plot of ^{131}I-hu4G4 to U87 cells. B: Specific Bound; B/F: Specific Bound/Reactive free.

Figure S3.

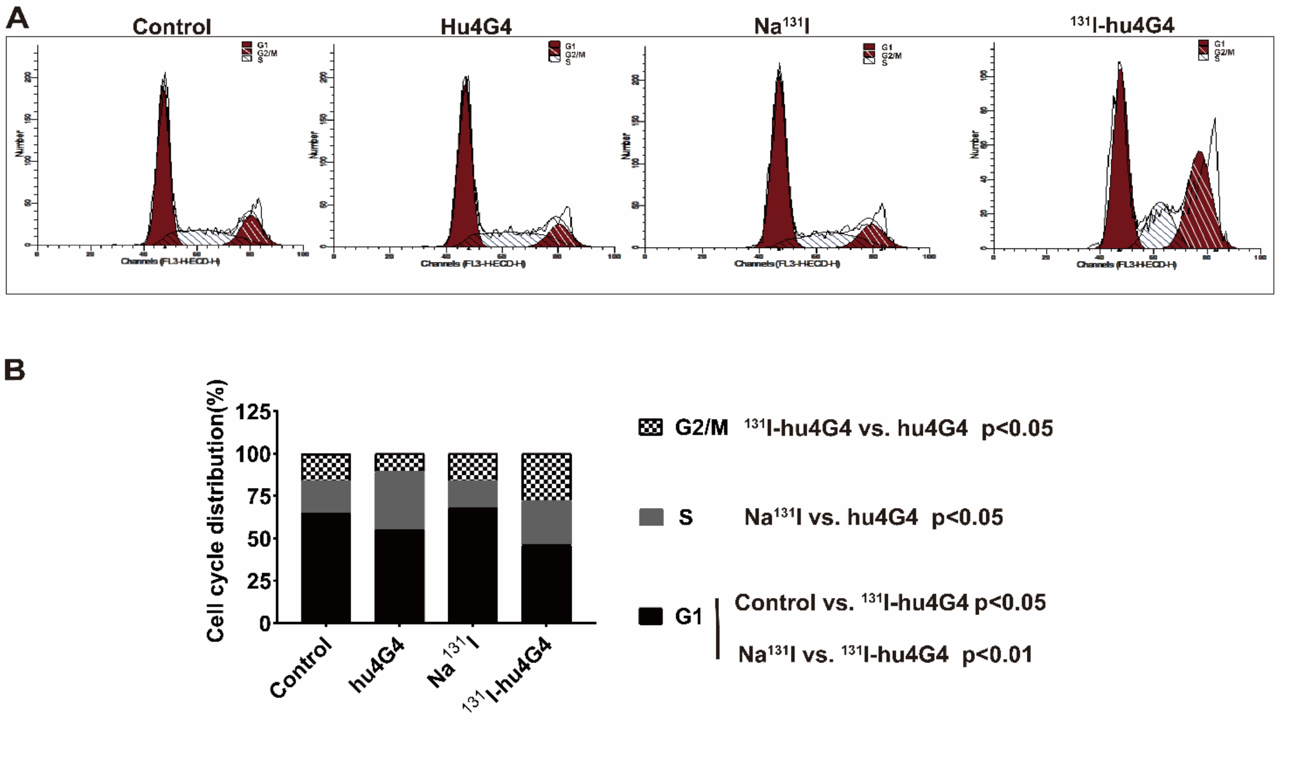

Figure S3. ^{131}I-hu4G4 induces G2/M phase arrest. (A) Cell cycle was detected by flow cytometry. (B) Quantitative results of cell-cycle assays found with U87 cells in each treatment group.

Figure S4.

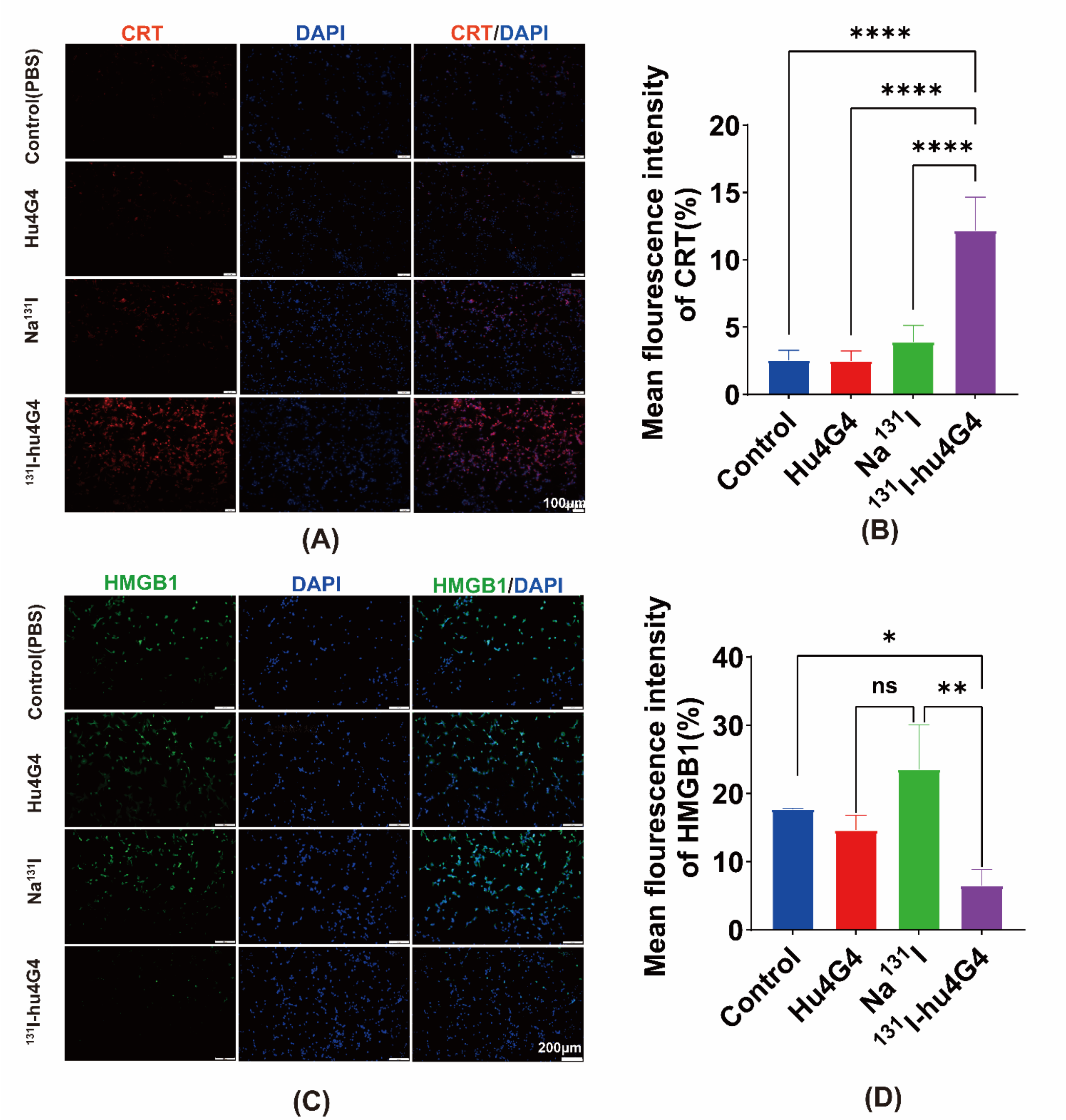

Figure S4. (A, C) Immunofluorescence-staining images showing CRT and HMGB1 levels in U87 cells at different treatment groups *in vitro*. (B, D) Mean fluorescence intensities for CRT and HMGB1 %.

Figure S5.

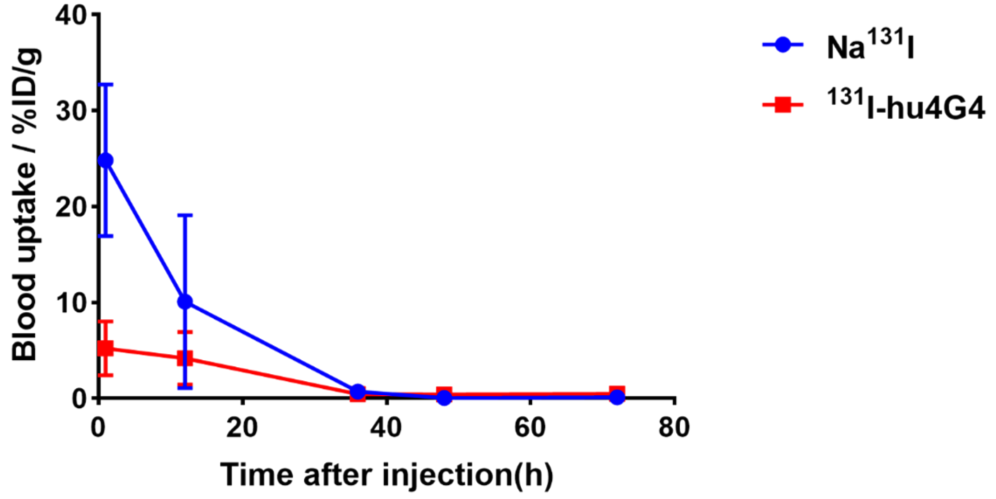

Figure S5. The blood uptake of ^{131}I-hu4G4 and Na^{131}I at different times (1, 12, 36, 48, and 72 h) after intracranial administration.

Figure S6.

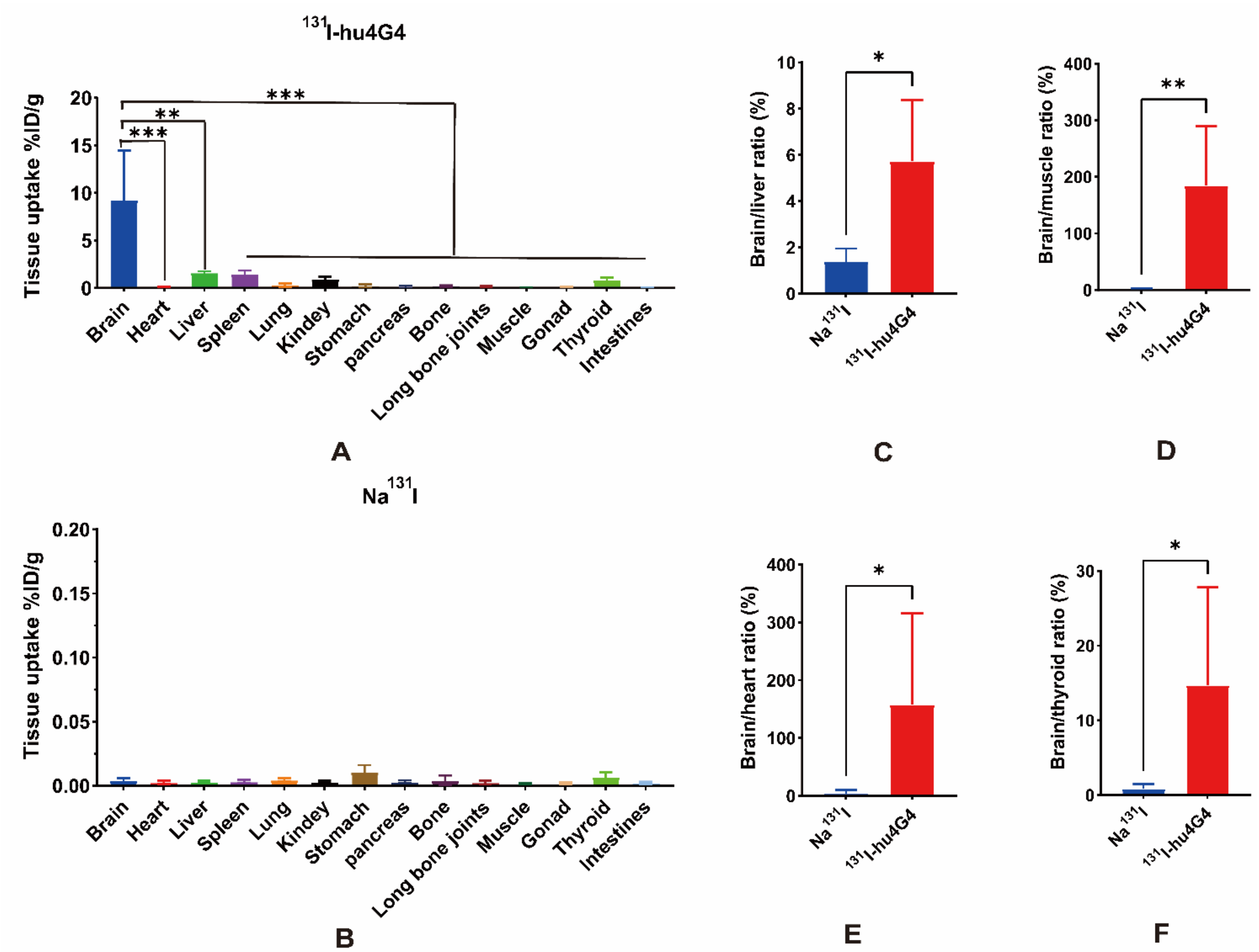

Figure S6. Biodistribution results of different tissues ex vivo (n=3). (A) Biodistribution results for the ^{131}I-hu4G4 group on day 7 after treatment. (B) Biodistribution results for the Na^{131}I group on day 7 after treatment. (C) Situ glioma tumor: liver ratios for the ^{131}I-hu4G4 and Na^{131}I groups on day 7 after treatment. (D) Situ glioma tumor: muscle ratios for the ^{131}I-hu4G4 and Na^{131}I groups on day 7 after treatment. (E) Situ glioma tumor: heart ratios for the ^{131}I-hu4G4 and Na^{131}I groups on day 7 after treatment. (F) Situ glioma tumor: thyroid ratios for the ^{131}I-hu4G4 and Na^{131}I groups on day 7 after treatment.

Figure S7.

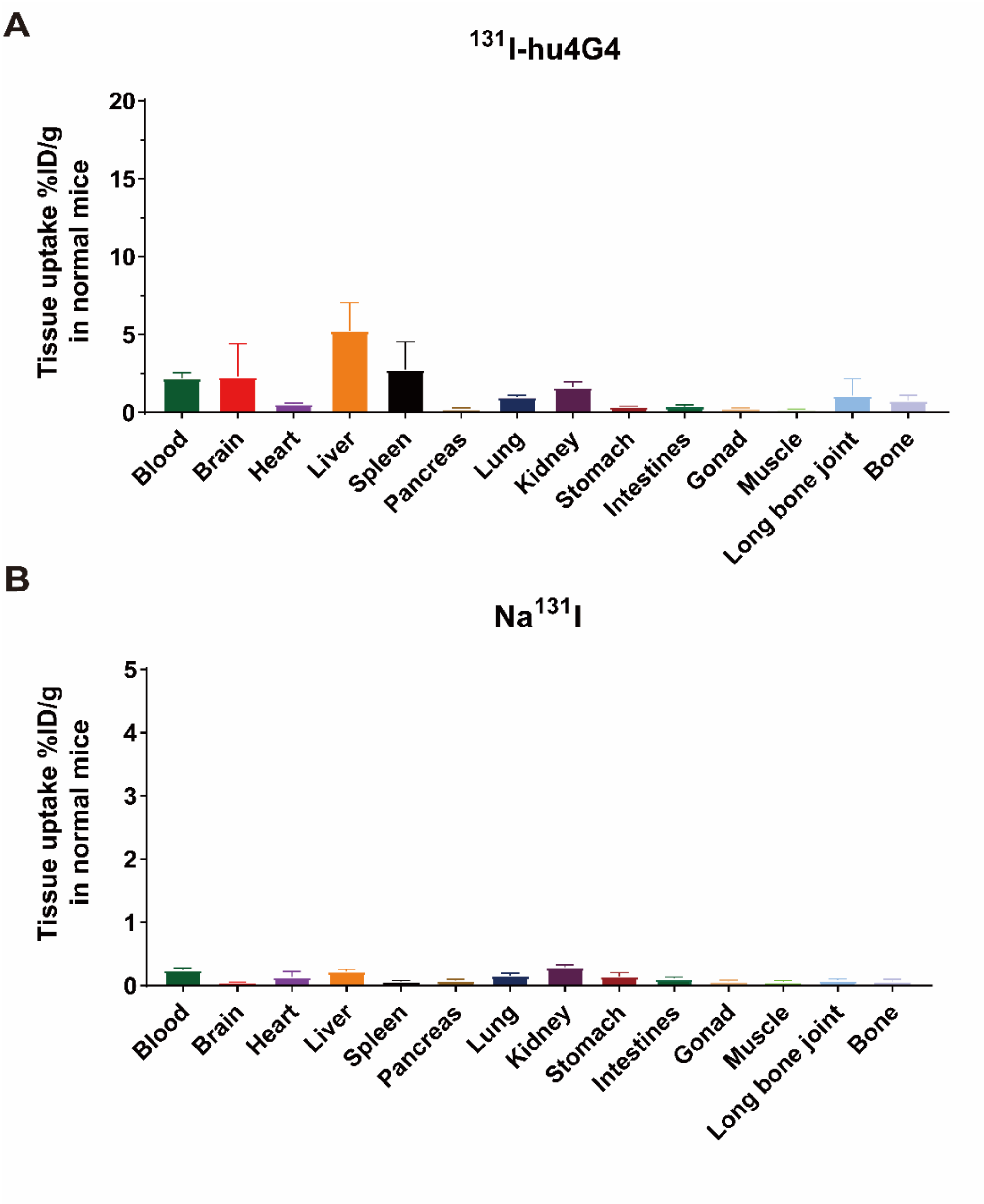

Figure S7. Biodistribution results of different tissues ex vivo (n=5). (A) Biodistribution results for the ^{131}I-hu4G4 group on day 7 after treatment in normal mice. (B) Biodistribution results for the Na^{131}I group on day 7 after treatment in normal mice.

Figure S8.

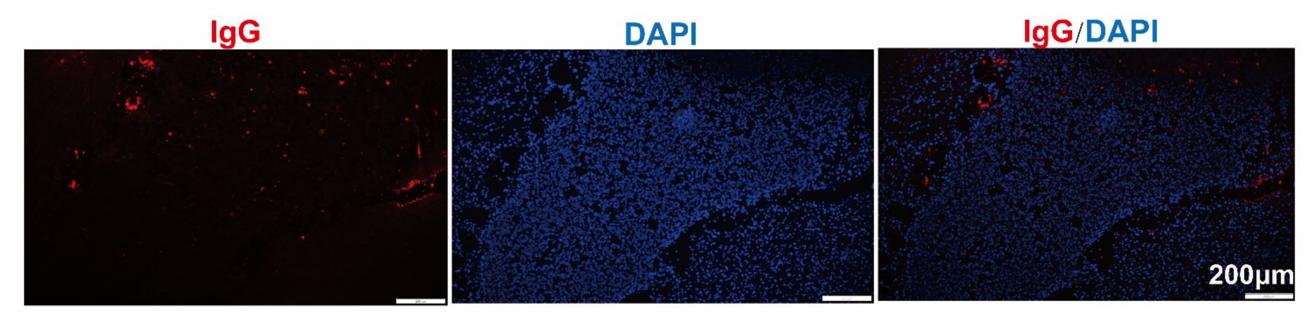

Figure S8. Immunofluorescence-staining images of IgG in brain glioma sections.

Figure S9.

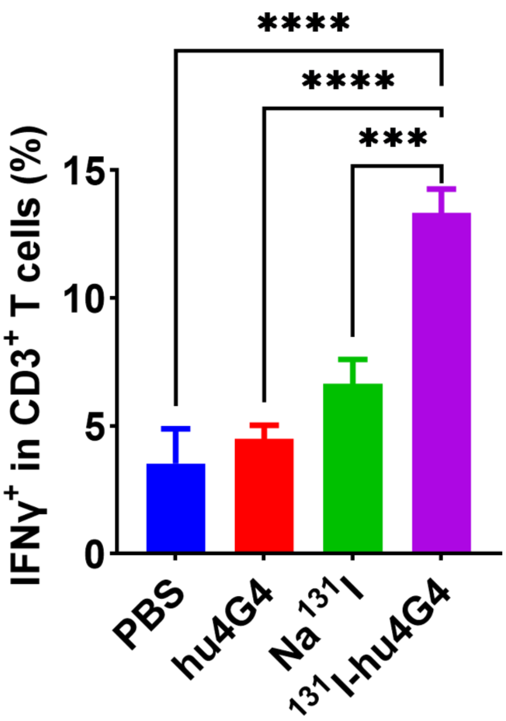

Figure S9. Tumors were surgically removed seven days following treatment. Tumor-derived single-cell suspensions were stained with IFN-γ antibodies, then flow cytometry was performed.
